# Functional Status and Quality of Life Determinants of a Group of Elderly People With Food Insecurity

**DOI:** 10.3389/fnut.2018.00099

**Published:** 2018-10-25

**Authors:** Frederico M. Baptista, Ana Maria Rodrigues, Maria João Gregório, Rute de Sousa, Eduardo Cruz, Helena Canhão

**Affiliations:** ^1^Escola Superior de Saúde, Instituto Politécnico de Setúbal, Setúbal, Portugal; ^2^CEDOC, EpiDoc Unit – Unidade de Epidemiologia em Doenças Crónicas, Nova Medical School, Lisbon, Portugal; ^3^Associação EpiSaúde, Évora, Portugal; ^4^Sociedade Portuguesa de Reumatologia, Lisbon, Portugal; ^5^Faculdade de Medicina da Universidade de Lisboa, Lisbon, Portugal; ^6^Faculdade de Ciências da Nutrição e Alimentação da Universidade do Porto, Porto, Portugal; ^7^Escola Nacional de Saúde Pública, Universidade Nova de Lisboa, Lisbon, Portugal

**Keywords:** elderly, food insecurity, functional status, functionality, quality of life

## Abstract

**Background:** A good functionality is appointed by the elderly as one of the most important factors for a good quality of life, since it is associated with independency and autonomy. Studies show that elderly with food insecurity have greater limitations in daily living activities (DLA) when compared to food security elderly. Moreover, food insecure elderly are 60% more likely to be diagnosed with depression. We aimed to investigate the potential determinants of functional status (HAQ) and quality of life (EQ-5D-3L) in a sample of elderly with food insecurity.

**Methods:** Forty-one participants with food insecurity were evaluated by a multidisciplinary team, in April and May of 2016 (a baseline cross-sectional study prior to the intervention program).

**Results:** This study demonstrates correlations of functional status and quality of life to such aspects of elderly assessment as age, BMI, manual strength, among others. It was found that manual strength, gender, family income, anxiety, and depression were correlated to quality of life; and that mobility, manual strength, anxiety and gender were correlated to the functional status. After multivariable adjustment, only mobility (β = −0.220; *p* ≤ 0.01) and quality of life (β = −1.457; *p* ≤ 0.01) remained significantly associated with higher levels of functional disability. With regard to quality of life, only the functional status (β = −0.242; *p* ≤ 0.01), the presence of depression (β = −0.169; *p* ≤ 0.05), and family income (β = 0.185; *p* ≤ 0.05) remained significantly associated with health-related quality of life.

**Conclusions:** The study aims to verify the potential determinants of functional status (HAQ) and quality of life (EQ-5D-3L) in a sample of elderly with food insecurity. Even in a small cohort, the study demonstrated that in an elderly population with food insecurity, functional status is associated with mobility and quality of life, and that health-related quality of life is also associated with symptoms of depression and family income. Larger studies in other populations may be useful to confirm these observations.

## Introduction

Food security is internationally defined as a situation that exists when all persons in the household, at all times have physical, social and economic access to sufficient, safe and nutritionally adequate food, to meet their nutritional needs and food preferences for an active and healthy life (1). On the other hand, Food Insecurity (FI) is characterized by a limited or uncertain access to adequate food in quantity and quality, by social and/or economic factors, preventing the maintenance of a healthy life (2).

Although it is more prevalent in low and middle-income countries, FI in households has also been identified and documented in high income countries (3).

In Portugal, data obtained through the EpiDoc cohort (2015–2016) in a representative sample of the Portuguese population, showed that 19.3% of the portuguese population had some level of FI, and that 1.8% were classified as having severe FI (4, 5). Among older adults, the prevalence of FI seems to be higher. Data from the same Portuguese cohort, showed that 23% of older adults were living in a food-insecure household. Moreover, FI in elderly is associated with higher prevalence of chronic diseases and decreased health-related quality of life (4, 5).

According to the Global Burden of Disease Study 2010's analyses, the dietary habits are the most detrimental to health and well-being in all countries of the European Union (6). FI has been associated with physical, psychological consequences, and has great impact in mortality, morbidity and quality of life (6). The elderly with FI are 60% more likely to be diagnosed with depression than the elderly with food security (7).

Some studies have shown that the elderly with FI have a greater probability of limitations in their daily living activities (DLA) compared to elderly people with food security (8). Among the aspects that are related to a good quality of life in old age, the elderly point to good functionality as one of the most important, since it is associated with independence and autonomy (9). Studies show that older adults who are able to maintain their functional mobility and independence, seem to be more likely to feel safer and have a better sense of well-being (10).

In summary, older adults find themselves in situations of greater vulnerability to adverse health conditions, with FI being a potential risk factor, with a considerable impact on muscle strength, mobility, autonomy, and mental health (11, 12).

The main objective of this study was to investigate, in a sample of elderly with FI, potential determinants of the functional status and quality of life of these elderly individuals, based on a cross-sectional analysis of the data obtained in an intervention study's baseline. Considering that the lack of valid and useful epidemiological information to support political decision-making is a major challenge for public health, this study aimed to contribute to a better knowledge and understanding of the potential determinants of functional status and the quality of life of a sample of elderly people with FI to allow the use of this information in the development of strategies to promote healthier lifestyles for the elderly.

## Materials and methods

### Study type

The research was carried out from the data collected at the baseline of the sample from Saúde.Come—senior—one of the intervention studies of “Saúde.Come—Promoting Food Security,” which was approved by the Ethics Committee of Nova Medical School/Faculty of Medical Sciences of Nova de Lisboa University (NMS/FCM-UNL) under number 20/2015/CEFCM. Details of the study protocol was published elsewhere (13).

All the individuals over 60 years old, who lived in their residence, who knew how to read, write and understand the Portuguese language, who were able to provide written consent, and who presented some level of FI (mild, moderate, or severe)—verified after the application of the portuguese version of the Food Insecurity Scale, were eligible for the study. Participants were recruited on a convenience basis from people who used the services of 17 health centers in the region of Lisbon and Vale do Tejo, between November of 2015 and March of 2016, in three sequential stages: 1—Screening to identify people with FI, from the application of the Food Insecurity Scale by a team of research assistant; 2—Invitation for a “baseline consultation,” to all eligible individuals; 3—Confirmation of inclusion/exclusion criteria, explanation of study objectives and procedures to participants, and written informed consent request. The evaluation of the participants integrated different parameters and dimensions of the social and health conditions and was carried out by a multidisciplinary team (physicians, physiotherapists, and nutritionists).

### Instruments of data collection

In this study, the following instruments were included for each variable of interest:

Sociodemographic and economic characterization questionnaire—it is included questions of sociodemographic characterization (age, gender, educational level, professional situation, and household status), and questions of economic characterization (family income).

Health Assessment Questionnaire (HAQ)—self-report questionnaire, to measure the functional status, whose score produces an “Incapacity Index.” It consists of 20 grouped questions into 8 dimensions including: dressing up and getting ready (2 questions), getting up (2 questions), eating (3 questions), walking (2 questions), personal hygiene (3 questions), reaching objects (2 questions), grabbing and opening objects (3 questions), and other day-to-day activities (3 questions). For each of these questions, the respondent indicates the degree of difficulty in 4 possible responses ranging from “no difficulty = 0” to “unable to do it = 3.” The instrument also includes a questionnaire on the use of assistive devices or the need for support from third parties to carry out the activities related to the 8 categories. The score for each category appears in the highest number of any of its items. The final HAQ score is the average score of 20 questions. The interpretation of the Functional State is performed according to the score obtained, considering the following classifications: HAQ of 0 to 1 = mild deficiency; HAQ >1 to 2 = moderate deficiency; e HAQ >2 to 3 = severe deficiency (14, 15).

European Quality of Life Questionnaire 5 Dimensions 3 Levels (EQ-5D-3L)—instrument for measuring health-related quality of life. It was designed to be self-filled by the interviewees. The instrument allows the junction of two components: (i) a profile describing the quality of life in terms of domains or dimensions; and (ii) a numerical value associated with the health status of the individual. The instrument contains 5 self-report dimensions (mobility, personal care, usual activities, pain/malaise, and anxiety/depression) with 3 levels of severity associated with each dimension: absence of health problems (level 1), some problems (level 2), and severe problems (level 3). These levels are identified according to the perception of the respondents (subjective data), and do not have arithmetic properties, thus, they should not be used as a cardinal score (16).

Hand Grip (Dynamometer CAMRY model EH 101) and Lafayette Manual Muscle Test System (model 01165)—instrument for measuring strength and manual grip. According to several authors, this is an important prerequisite for the identification of upper limb functional conditions (17).

Elderly Mobility Scale (EMS)—it is a scale oriented toward assessing the performance and mobility of frail elderly. It has 7 items grouped in 2 subscales (dimensions). The first subscale (EMS—BT) is composed of 2 items related to mobility/bed transfer (“lying down to standing up” and “standing up to lying down”) and the second subscale (EMS—FM) has 5 items related to functional mobility (“sitting up” to “standing up,” “standing,” “walking,” “timed walking,” and “functional range”). The maximum score for each item on the first subscale is 2. The maximum score for the first four items of the second subscale is 3, while the maximum score for the last item of the second subscale is 4. The higher the score, the greater the degree of independence/mobility of the respondent. The final interpretation of the score obtained can be made according to the following classification: ≥14 points = elderly with good mobility and balance; 6–13 points = elderly people who may need some extra help to be able to walk independently; and <6 points = individuals with poor mobility, that is, they can independently perform only some changes of position in the bed (18).

Hospital Anxiety and Depression Scale (HADS)—it is a self-assessment scale consisting of 14 items (7 items to assess anxiety and 7 items to assess depression), to briefly assess anxiety levels (HADS-A) and depression (HADS-D). For each item there are four possibilities of response, in a Likert scale of 4 points, the person should choose the one that adapts to the way they have felt during the last week. The response possibilities range from “0” (low) to “3” (high). The total results for each subscale (HADS-A e HADS-D) range from 0 to 21, resulting from the sum of the values of the items in each subscale, with higher values indicating high levels of anxiety and depression. This scale was culturally adapted and validated for Portuguese by Pais-Ribeiro et al. (19), showing similar psychometric properties to the original version. The scores can be interpreted considering the following cut-off point: presence of anxiety symptoms (HADS-A ≥11 vs. <11); and presence of depression symptoms (HADS-D ≥11 vs. <11) (19).

### Procedure for collecting data

The data collection relating to the instrument's evaluation used in this study occurred in three sequential moments: (1) medical appointment; (2) physiotherapist consultation; (3) nutritional appointment. At the medical consultation, data were collected on the identification and contact of the participants, it was obtained socio-economic information and questionnaires were applied on Quality of Life (EQ-5D-3L), Functional Status (HAQ), and Anxiety and Depression Status (HADS), respectively. The physiotherapy evaluation consisted of examining manual grip muscle strength, knee extension and hip flexion using a dynamometer and the Lafayette Manual Muscle Test System. The physiotherapist also applied the EMI. The anthropometric data that allowed the calculation of body mass index (BMI) were collected at the nutritionist's consultation.

### Data analysis

Data analysis was performed using the SPSS statistical program (Statistics Package for the Social Sciences—version 24).

The sociodemographic characteristics were systematized through descriptive statistics, using the relative frequencies when the variables were nominal/categorical. For continuous variables—functional status, quality of life, age, and BMI—we used measures of central tendency (means) and dispersion measures (standard deviation and maximum and minimum intervals).

To identify associations between the potential determinants factors and the dependent variables—functional status (HAQ) and health-related quality of life (EQ-5D-3L)—a bivariate analysis was performed. Prior to the correlation analysis, normality in the distribution of the variables was verified through the Shapiro-Wilk test. As there was no normality regarding the distribution of variables (only a normal distribution in the muscle strength variable being notorious), we chose to use the Spearman correlation test to analyze the associations between variables (20). It was conventionally used that *r* < 0.2 indicates a very low association; between (0.2; 0.39) low; between (0.40; 0.69) moderate; between (0.70; 0.89) elevated; and finally, between (0.9; 1) a very high association (21).

In order to explore the relationship between qualitative variables and functional status and quality of life, it was firstly transformed into dichotomous variables. Thus, the levels of the variable “Professional Situation” were aggregated into “active (part-time or full-time)/non-active (domestic, retired and unemployed),” the levels of the variable “Household” in “live alone (single, widowed, or divorced)/live together (married),” and the levels of “Family income,” in “ ≤1,000 euros/>1,000 euros.” Levels of the “FI” variable was aggregated into “mild/moderate FI or severe FI.” Finally, HADS scores for anxiety and depression were aggregated according to cut-off points provided in the literature for this instrument to identify the presence/absence of anxiety symptoms (HADS-A ≥11 vs. <11), and the presence/absence of symptoms of depression (HADS-D ≥11 vs. <11) (19, 22).

The correlational analysis of these variables with the continuous variables was performed using the bi-serial point correlation coefficient. The assumptions to perform this procedure were previously verified, not confirming the presence of outliers in any of the variables under the study. Through the Levene test, it was possible to confirm the homogeneity of the variances for the functional status and the quality of life, for all variables under the study (*p* > 0.05). The same was observed with the normal distribution of the variables (except for the professional situation levels).

The bivariate analysis allowed to identify which variables to consider in the regression model. Considering the low number of participants in the study sample, the reduction in the number of independent variables favored the statistical power of the regression analysis. Thus, in order to investigate the relative contribution of the independent variables to the explained variance of functional status and quality of life, statistically significant variables were submitted to multiple linear regression using the Backward selection method.

For the tests performed, the lower limit of significance was set at *p* < 0.05 (95% confidence level), and the null hypothesis was rejected when the probability of significance of the test (*p*-value) was lower than this value.

## Results

**Figure 1:**
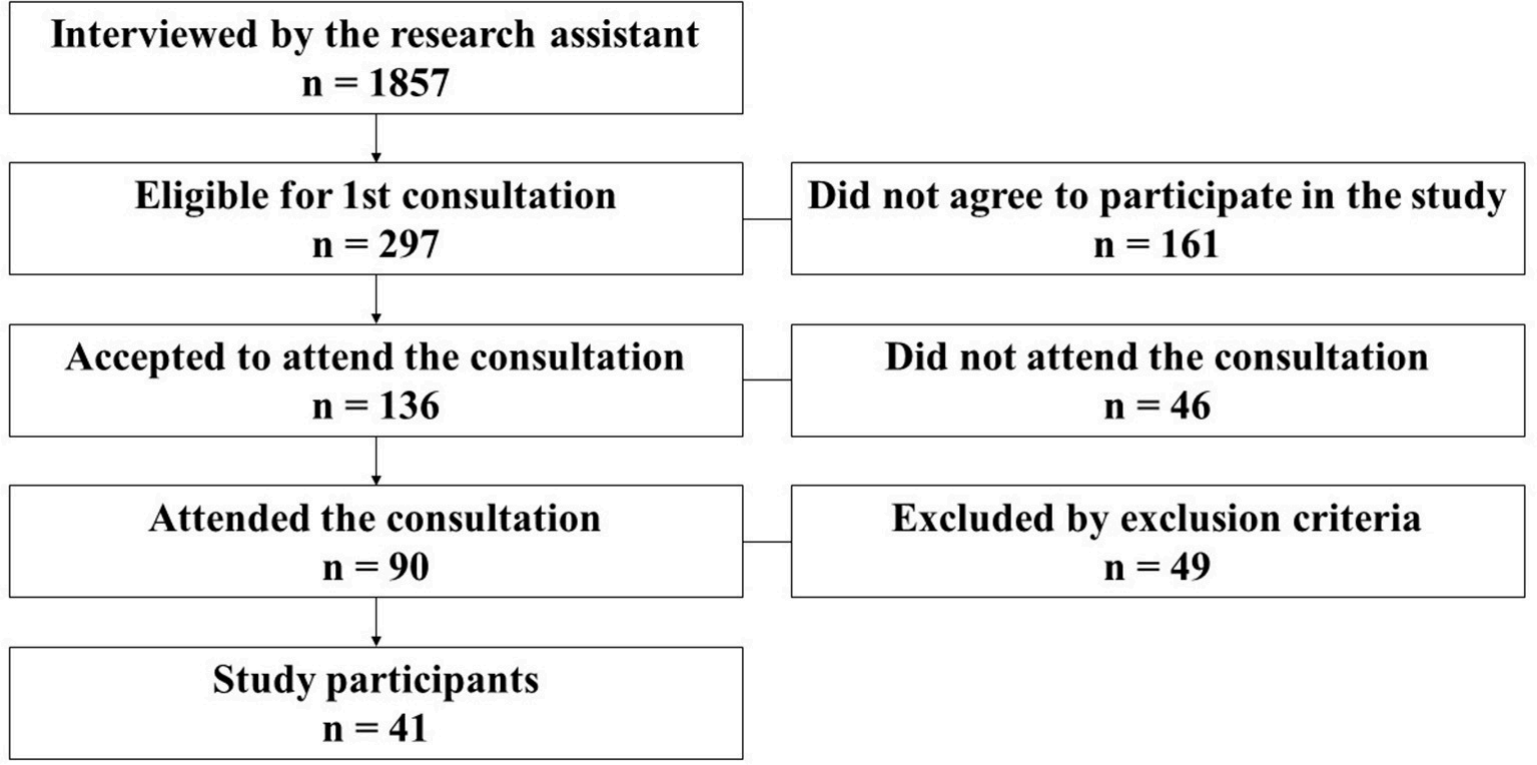
Study flowchart.

Of a total of 1,857 people interviewed by the research assistant team at health centers, 297 were considered eligible to go to the 1st consultation (baseline consultation) at the same health centers where they were previously recruited. Of these, 161 people did not agree to make appointments or were not available to participate in the study, and 136 people had a marked appointment, of which only 90 attended. Of the 90 participants, 49 were excluded because they had not been confirmed by a nutritionist, as having FI after fulfilling the Food Insecurity Scale, or because they did not meet any of the inclusion criteria established for the study, thus, the total number of participants in this study was 41 (Figure 1).

The sociodemographic characteristics of the sample are specified according to Table 1.

**Table 1:** Sociodemographic characteristics of the sample.

**Variable in analysis**	**Variable categories**	**Fr**^**a**^**/descriptive statistic**	
Age (Mean ±*SD*)	–	69.73 years (±6.36 years)	
Age (minimum and maximum)	–	60 years	82 years
BMI (Mean ±*SD*)	–	30.82 kg/m^2^ (±5.94 kg/m^2^)	
Gender	Male	31.7% (*n* = 13)	
	Female	68.3 % (*n* = 28)	
Occupational status	Without working	95.1% (*n* = 39)	
	Working	4.9% (*n* = 2)	
Family income (month)	≤1,000 euros	85.4% (*n* = 35)	
	>1,000 euros	14.6% (*n* = 6)	
Household	Live alone	39% (*n* = 16)	
	Live together	61% (*n* = 25)	

Regarding the Functional Status (HAQ) of the participants and considering the Functional Status Incapacity Index (HAQ), the sample had an average value of 0.78 (± 0.65) in the HAQ score (0–3).

In regard to the characterization of the quality of life of the participants, the sample had a mean value index of 0.58 (±0.27) converted from the descriptive system of the EQ-5D-3L instrument (Table 2).

**Table 2:** Characteristics of the sample.

**Variable in analysis**	**Variable categories**	**Fr^a^/descriptive statistic**
HAQ (Mean ±*SD*)	–	0.78 (±0.65)
EQ-5D-3L (Mean ±*SD*)	–	0.58 (±0.27)
Manual strength (Mean ±*SD*)	–	25.09 lbs (±6.88)
Knee extension strength average (Mean ±*SD*)	–	17.01 lbs (±7.08)
Hip flexion strength average (Mean ±*SD*)	–	16.90 lbs (±7.58)
EMS (Mean ±*SD*)	–	19.46 (±1.07)
HADS-A	Absence of anxiety symptoms (HADS-A <11)	63.4%
	Presence of anxiety symptoms (HADS-A ≥11)	36.6%
HADS-D	Absence of depression symptoms (HADS-D <11)	85.4%
	Presence of depression symptoms (HADS-D ≥11)	14.6%

### Bivariate correlations of the functional status and quality of life with the independent variables

In relation to the Functional Status (HAQ) and considering that the score in the HAQ increases with the disability, the associations with Quality of Life (EQ-5D-3L) (*p* ≤ 0.001), mobility (EMS) (*p* ≤ 0.001), and manual strength (*p* ≤ 0.01) were statistically significant (Table 3). The analysis of the Spearman's rho (correlation coefficients) allows us to verify that: (1) there is a negative and moderate correlation between the Quality of Life (EQ-5D-3L) and the Functional Status (HAQ); (2) there is a negative and moderate correlation between Mobility (EMI) and Functional Status (HAQ); and (3) there is a negative and low correlation between the manual grip force and the Functional Status (HAQ). Regarding Quality of Life (EQ-5D-3L), there was also a positive, significant, and low correlation with the manual force (rho = 0.381, *p* ≤ 0.01) in addition to Functional Status (Table 3). With regard to the results of the bi-serial correlational analysis between the dichotomous variables and the Functional Status (HAQ), there was a negative, significant and weak correlation with gender (rpb = −0.346, *p* ≤ 0.05), and a positive, significant and moderate correlation with anxiety (HADS-A) (rpb = 0.414, *p* ≤ 0.01; Table 3). In the analysis between dichotomous variables and Quality of Life (EQ-5D-3L), there was a positive, significant and weak correlation with gender (rpb = 0.332, *p* ≤ 0.05), a positive, significant, and weak correlation with family income (rpb = 0.366, *p* ≤ 0.05), and negative, significant, and weak correlations with anxiety (HADS-A) (rpb = −0.357, *p* ≤ 0.05) and with depression (HADS-D) (rpb = −0.372, *p* ≤ 0.05; Table 3).

**Table 3:** Bivariate Analysis of Quality of Life (EQ-5D-3L) and Functional Status (HAQ) with the independent variables.

	**Quality of life (EQ-5D-3L)**				**Functional status (HAQ)**			
	**Spearman coefficient**	***p-value***	**Pearson coefficient**	***p-value***	**Spearman coefficient**	***p-value***	**Pearson coefficient**	***p-value***
Age	−0.167	0.302	–	–	0.120	0.455	–	–
BMI	−0.173	0.292	–	–	0.289	0.070	–	–
Quality of life (EQ-5D-3L)	1.000	–	–	–	−0.666^**^	0.000	–	–
Functional status (HAQ)	0.666^**^	0.000	–	–	1.000	–	–	–
Mobility (EMS)	0.299	0.061	–	–	−0.509^**^	0.001	–	–
Manual strength	0.381^*^	0.017	–	–	−0.369^*^	0.019	–	–
Knee extension strength average	0.168	0.300	–	–	−0.254	0.109	–	–
Hip flexion strength average	0.101	0.541	–	–	−0.148	0.362	–	–
FI (Food insecurity score)	−0.160	0.324	–	–	0.178	0.265	–	–
Women	–	–	0.332^*^	0.037	–	–	−0.346^*^	0.027
Occupational status^*^^a^	–	–	0.029	0.860	–	–	−0.164	0.307
Family income^*^^b^	–	–	0.366^*^	0.020	–	–	−0.219	0.169
Household^*^^c^	–	–	0.060	0.715	–	–	−0.040	0.806
Anxiety (HADS-A)	–	–	−0.357^*^	0.024	–	–	0.414^**^	0.007
Depression (HADS-D)	–	–	−0.372^*^	0.018	–	–	0.260	0.101

* The correlation is significant at the 0.05 level (2 extremities).

** The correlation is significant at the 0.01 level (2 extremities).

a Without working vs. working.

b ≤ 1,000 euros vs. >1,000 euros.

c *Live alone vs. live together*.

### Multivariate analysis

Variables with statistical significance in relation to dependent variables, functional status and health-related quality of life, were included in the multiple linear regression, in order to identify the relative contribution of each to the dependent variables.

For the analysis of multivariate relationships, with adjustment for confounders, two linear regression models were used (Table 3). In the simultaneous evaluation of the variables, gender, hand grip strength and presence of anxiety lost their influence, with only mobility (*p* ≤ 0.01; β = −0.362) and quality of life (*p* ≤ 0.01; β = −0.610) as significantly influential for higher levels of functional disability, accounting for 61.1% of their variability (Table 4).

**Table 4:** Factors associated with the Functional Status (HAQ) and with the Quality of Life (EQ-5D-3L): Multivariate Model.

**Independent variables**	**Functional status (HAQ)**			**Quality of life (EQ-5D)**		
	**β**	**(95% IC)**	***p-value***	**β**	**(95% IC)**	***p-value***
Quality of life (EQ-5D-3L)	−1.457^*^	(−1.969; −0.945)	0.000	–	–	–
Anxiety (HADS-A)	0.036	(−0.298; 0.394)	0.778	−0.022	(-0.160; 0.136)	0.869
Gender (female)	−0.084	(−0.427; 0.193)	0.448	0.029	(-0.126; 0.159)	0.813
Mobility (EMS)	−0.220^*^	(−0.350; −0.089)	0.002	–	–	–
Manual strength	0.008	(−0.023; 0.039)	0.596	–	–	–
Functional status (HAQ)	–	–	–	−0.242^*^	(−0.339; −0.145)	0.000
Family income	–	–	–	0.185^*^	(0.013; 0.356)	0.035
Depression (HADS-D)	–	–	–	−0.169^*^	(−0.343; 0.004)	0.055

DEPENDENT variable: Functional Status (HAQ) and Quality of Life (EQ-5D-3L).

* *Adjusted p < 0.05 EQ-5D-3L – European Quality of Life Questionnaire Five Dimensions Three Levels; HADS, Hospital Anxiety and Depression Scale; EMS, Elderly Mobility Scale; HAQ, Health Assessment Questionnaire*.

Regarding the quality of life (EQ-5D-3L), in the simultaneous evaluation of the variables, gender and the presence of anxiety lost their influence, being only the functional status (β = −0.242, *p* ≤ 0.01), the presence of depression (β = −0.169, *p* ≤ 0.05), and family income (β = 0.185, *p* ≤ 0.05), as significantly influential for quality of life, which together account for 54.4% of its variability (Table 4).

## Discussion

The study verified that, regarding the quality of life (EQ-5D-3L score) of study participants (0.5 ± 0.2), the sample presented worse results when compared to the data obtained with the use of the same instrument in a population-wide epidemiological study (EpiReumaPt) performed with a sample of the Portuguese (not necessarily elderly) adult population, published in 2016, where the mean score was 0.7 (±0.3) for individuals with some rheumatic and/or musculoskeletal pathology, and 0.9 (±0.1) for individuals without the presence of any rheumatic and/or musculoskeletal pathology (23).

Regarding the functional status (HAQ) of the participants (0.7 ± 0.6), the sample also presented worse results regarding the EpiReumaPt study, where the mean score obtained in the HAQ was 0.4 ± 0.7 for individuals with any rheumatologic or musculoskeletal condition, and 0.1 ± 0.2 for subjects without any of these conditions (23).

Thus, through the comparison of these results, although the EpiReumaPt study was performed with a sample with different characteristics from that of this study, it would be possible, however, to hypothesize that FI associated with older age could be a risk factor for increased disability, as well as for a lower quality of life among elderly individuals with FI.

However, this study did not demonstrate a statistically significant correlation of the dependent variables (HAQ and EQ-5D-3L) with the Food Insecurity Scale score, which may be explained by the small sample size.

Regarding the association of the functional status with the other verified variables it was found that, in relation to muscle strength, the data obtained in the multivariate analysis did not confirm findings from previous studies that found a significant correlation between functional capacity and some physical-functional attributes in the elderly, such as muscle quality (ratio of muscle mass to muscle strength), strength and muscle power (24–26).

Other factors associated with impaired functional capacity in the elderly described in the literature include dimensions of mental health, such as cognitive decline and the presence of depression (9), which was also not verified in the correlational analyzes of the present study.

In fact, the results showed a significant correlation between functional status and mobility and the quality of life of the elderly constituents of this sample, which reaffirms findings already described in the literature that show that, among elderly at nutritional risk, functional limitations, are negatively associated with quality of life (27), and that functional disability has in turn been associated with mobility deficits, particularly in older adults (28).

Regarding quality of life, correlational analyzes showed a significant correlation of this variable with functional status, symptoms of depression and family income, which reaffirms findings already described in the literature (9, 27, 28). Regarding the association between the quality of life and the mobility of the elderly participants, there was no significant association between these two variables, and it was not possible to confirm the findings related to the study by Davis et al. (10). In fact, there was a ceiling effect in the results obtained with the application of EMS in this study, that is, there was an accumulation of scores at the upper level reported by the instrument. In this way, it was not possible to accurately distinguish the degree of mobility of the interviewees above the highest level obtained by the research instrument. For later studies, with similar samples to that of this research, it is recommended to use another instrument to evaluate the mobility of the elderly.

The study presents some limitations, such as the fact that the measurement of most variables (with the exception of muscle strength and mobility) was performed through the use of self-report instruments, which allows a greater occurrence of measurement errors, since other (uncontrolled) factors may have influenced the way people answered the questions. Moreover, the results found may not accurately represent the reality of the elderly population with FI due to the small size of the sample. Also, considering that the study did not include a control group of participants who have not been food insecure, there is a probability that all the found correlations may also appear in food secure elderly population, meaning that it is not possible to confirm that these findings are specific for food insecurity. Therefore, it may be useful to carry out further larger studies including control groups, and in other populations, in order to confirm the observations found.

Another relevant aspect of highlighting here concerns the cross-sectional nature of the study. It is important to emphasize that the results obtained do not show any cause and effect relationship between the variables. It only shows a probable association between them. Considering that there is a lack of valid and useful epidemiological data to facilitate the development of social programs aimed at the elderly population with the aim of improving their nutritional balance, the functionality, and quality of life, this study aimed to contribute to a better knowledge and understanding of the potential determinants of the functional status and quality of life of the elderly with FI, in order to facilitate the development of strategies to promote healthier lifestyles and maximize health gains. In conclusion, the present study demonstrated that in a particularly vulnerable elderly population and with FI, functional status was shown to be associated with mobility and quality of life, and that quality of life, in turn, has been shown to be associated, in addition to functional status, to depression symptoms and to the family income of the elderly participants in the study. Considering the results found, it is recommended to carry out other longitudinal and/or experimental studies in order to: 1—more accurately examine the potential determinants of functional status and quality of life in an elderly population identified with some level of FI; and 2—explore the use of more appropriate instruments for the evaluation of variables in larger samples.

The importance and relevance of this study lie in the fact that few studies have been published at European level regarding the identification of possible factors determining the functional status and quality of life of elderly people with food insecurity. Thus, this study serves as a starting point for new researches with larger samples and with less limitations to be made so that, in fact, we can identify such factors, in order to facilitate the elaboration of strategies to improve the quality of life and functional status of food-insecure elderly.

## Author contributions

All authors collected data for the study. HC, AR, and RdS supported the development of the study design and methodology. FB conducted the study and wrote the paper. HC and EC supported the whole development of the study and writing and reviewed the paper. EC supported the data analysis process.

### Conflict of interest statement

The authors declare that the research was conducted in the absence of any commercial or financial relationships that could be construed as a potential conflict of interest.

## Abbreviations

BMI: body mass index
DLA: daily living activities
EMS: Elderly Mobility Scale
EQ-5D-3L: European Quality of Life Five Dimensions Three Levels
FI: food insecurity
HADS: Hospital Anxiety and Depression Scale
HAQ: Health Assessment Questionnaire.
